# Predictors of intraoperative and postoperative air leakage in anatomical pulmonary resection

**DOI:** 10.1007/s00595-025-03004-2

**Published:** 2025-03-03

**Authors:** Jotaro Yusa, Kazuhisa Tanaka, Yuki Sata, Takahide Toyoda, Terunaga Inage, Hajime Tamura, Masako Chiyo, Yukiko Matsui, Ichiro Yoshino, Hidemi Suzuki

**Affiliations:** 1 Department of General Thoracic Surgery, Chiba University Graduate School of Medicine, 1-8-1, Inohana, Chiba 260-8670 Japan; 2 Department of Thoracic Surgery, International University Health and Welfare School of Medicine, Narita, Japan

**Keywords:** Air leakage, Stapler, Anatomical pulmonary resection

## Abstract

**Purpose:**

Air leakage is a common complication of lung surgery that sometimes requires medical intervention and leads to a prolonged hospital stay. This study assessed the risk factors associated with air leakage following anatomical pulmonary resection.

**Methods:**

We retrospectively analyzed 194 patients who underwent anatomical pulmonary resection in 2020. A risk factor analysis was performed separately for intraoperative air leakage (IAL) and postoperative air leakage (PAL) by examining patient characteristics and operative findings.

**Results:**

Of 194 patients, IAL was observed in 94 (48.4%). The number of staplers used for pulmonary resection and Brinkman index were significantly higher in the IAL group than in the non-IAL group (3.2 vs. 2.5, p = 0.005 and 696.2 vs. 477.1, p = 0.013, respectively). PAL was observed in 40 (20.6%) patients (25 IAL and 15 non-IAL patients). There were more lobectomy cases in the PAL group than in the non-PAL group (77.5 vs 60.4%, p = 0.0447). Pleurodesis was performed in 18 (45%) patients in the PAL group.

**Conclusion:**

The risk factors differed between IAL and PAL. Since most PAL cases involve the IAL, and approximately half of the PAL cases require pleurodesis, attention should be paid to PAL prevention, especially during stapling procedures in the lung parenchyma.

## Introduction

Air leakage following pulmonary resection is a common complication encountered in thoracic surgery [1, 2]. It is predominantly attributed to intraoperative surgical trauma or manipulation of the lung parenchyma [3]. Intraoperative air leakage (IAL), which is caused by operative procedures and is recognized by an air-leak test immediately before the end of an operation, typically arises at sites of adhesion dissection, lung handling, or along the staple line of mechanical suturing devices [4]. Major IAL must be repaired intraoperatively, which prolongs operative time. In addition to intraoperative factors, patient-specific factors such as emphysema, chronic obstructive pulmonary disease (COPD), interstitial pneumonia (IP), diabetes mellitus, and steroid use have been implicated in the development of postoperative air leakage (PAL). In addition, the type of surgical procedure and remaining intrapleural space after pulmonary resection are recognized as significant contributors [5–11]. Although several studies have explored the risk factors associated with prolonged air leakage, few have focused on the role of staplers [4].

This study evaluated the factors that contribute to IAL and PAL, and their relationship in patients undergoing anatomical pulmonary resection.

## Methods

### Patient selection

This retrospective study was conducted in accordance with the 1964 Declaration of Helsinki and its amendments. This study was approved by the Ethics Committee of Chiba University Graduate School of Medicine (M10271), and an opt-out method of informed consent was used.

We reviewed the medical and surgical records of 383 patients who underwent pulmonary resection at the Department of General Thoracic Surgery, Chiba University Hospital, between January 2020 and December 2020.

**Fig. 1:**
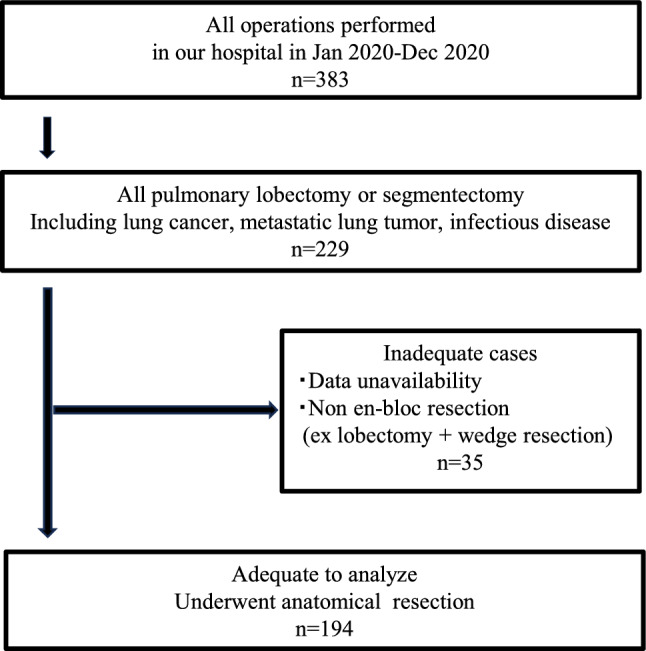
Flowchart of patient selection

Of these, 229 underwent anatomical pulmonary resection. To analyze the risk factors for IAL and PAL, 194 patients who underwent simple lobectomy or segmentectomy with en bloc resection were included. Patients with missing data or those who had undergone pneumonectomy, bilobectomy, or multiple pulmonary resections were excluded (Fig. 1).

An automatic suturing device is often used during lung parenchymal resection in segmentectomy, and a buttress device is sometimes used when the lung is likely to be fragile.

All patients underwent a routine preoperative checkup with chest radiography, computed tomography (CT) of the chest, electrocardiography, and pulmonary function tests. Other examinations, such as ^18^F-fluorodeoxyglucose-positron emission tomography, magnetic resonance imaging, and a cardiac evaluation with sonography, were performed when necessary.

Patient background information, including the sex, age, smoking history, steroid use, comorbidities (e.g. COPD and IP), medical history, respiratory function, clinical diagnosis, clinical course (presence of PAL and PAL requiring pleurodesis), duration of chest drain placement, and hospitalization, were extracted from the medical records [12]. COPD was defined as a forced expiratory volume-to-forced vital capacity (FEV/FVC) ratio of < 70%, whereas IP was diagnosed based on the serum KL-6 level and preoperative CT findings. Surgical records were examined to determine the surgical procedure, operative approach, operative time, blood loss, number of staplers used for pulmonary resection, and the presence of IAL.

### Air leakage

The presence of IAL was assessed using a water-sealing test immediately before chest closure. If an IAL was detected, it was repaired before surgery was completed. Air leakages detected during surgery were repaired using lung sutures with polyglycolic acid sheets or fibrin glue applied to reinforce the closure at the pleural defect sites. Repair methods were consistent across cases, and no significant differences were observed between PAL and non-PAL outcomes. In almost all cases, the repair was reinforced by placing a pericardial fat pad on the bronchial stump and spraying fibrin glue. In some cases, the lungs were sprayed to prevent air leakage simultaneously; however, the medical records did not clearly indicate the number of patients who underwent this procedure.

Chest drainage tubes were managed according to a clinical protocol, with either a traditional drainage system based on a three-bottle system or a digital drainage system (Thopaz^®^; Medela, Tokyo, Japan). Air leakage was monitored postoperatively, and the chest drainage tube was removed once lung expansion was confirmed, air leakage was absent, and the drainage volume was < 200 ml/day or expected to fall below 200 ml/day.

PAL was evaluated based on the presence of postoperative air leakage. Data were extracted from postoperative medical records and were primarily assessed from the first postoperative day. If air leakage persisted and was deemed intractable, pleurodesis was generally performed within four days after surgery.

Owing to the spread of severe acute respiratory syndrome coronavirus 2 (SARS-CoV-2) in 2020, early discharge was prioritized to minimize the risk of infection. Therefore, pleurodesis was implemented to reduce the length of hospital stay in cases of postoperative air leakage. In brief, 5 KE of OK-432 (Picibanil^®^; Chugai Pharmaceutical, Tokyo, Japan) was injected into the thoracic cavity through the chest tube, and the patient was placed in a supine position at rest with the drainage tube elevated over the bed rail to stop drainage for 1 h. Chest tube removal was performed as previously described.

### Statistical analyses

Continuous variables are presented as means and standard deviations, and categorical variables are presented as frequencies and percentages. Categorical variables were compared using Pearson’s chi-square test, whereas continuous variables were compared using Student’s *t*-test. All tests were two-tailed, and statistical significance was set at P < 0.05. Statistical analyses were performed using the SAS statistical software package, version 9.4 (SAS Institute, Cary, NC, USA).

## Results

The baseline characteristics of 194 patients are shown in Table 1. The mean patient age was 69.3 (range, 29–88) years old, and the mean Brinkman index was 580.2. Sixty-eight patients (35.0%) had COPD, and 22 (11.3%) had IP as a comorbidity. Lobectomy was performed in 124 patients (63.9%), and segmentectomy was performed in 70 patients (36.1%). Surgical approaches included robot-assisted thoracic surgery (RATS) in 32 patients, video-assisted thoracic surgery (VATS) in 22, and open thoracotomy in 139. The mean operative time was 164 (range, 56–476) min, and the mean blood loss was 84 (range, < 10–1755) ml. A mean of 2.85 (range, 0–8) staples were used for pulmonary resection. The mean duration of chest drainage was 2.56 (range, 1–7) days, and the mean hospital stay was 10 (range, 4–115) days. IAL occurred in 94 (48.6%) patients, of whom 25 (26.6%) developed PAL.

**Table 1:** Patient characteristics

Variables	n = 194
Age (SD)	69.3 (9.6)
Sex	
Male (%)	124 (63.9)
Female (%)	70 (36.1)
Brinkman index (SD)	580.2 (592.7)
DM (%)	34 (17.5)
Steroid use (%)	12 (6.18)
COPD (%)	68 (35.0)
IP (%)	22 (11.3)
Approach (%)	
RATS	33 (17.0)
VATS	22 (11.3)
Other	139 (71.7)
Procedure, n (%)	
Lobectomy	124 (63.9)
Segmentectomy	70 (36.1)
Operation time (min) (SD)	164 (56)
Bleeding (ml) (SD)	85 (196)
Staplers used for pulmonary resection (SD)	2.85 (1.62)
Duration of chest tube placement (days) (SD)	2.56 (1.26)
Length of hospitalization (days) (SD)	10.0 (9.11)

Data are shown as the mean (standard deviation) or number (percentage)

DM: diabetes mellitus; COPD: chronic obstructive pulmonary disease; IP: interstitial pneumonia; RATS: robot-assisted thoracic surgery; VATS: video-assisted thoracic surgery; SD: standard deviation

**Fig. 2:**
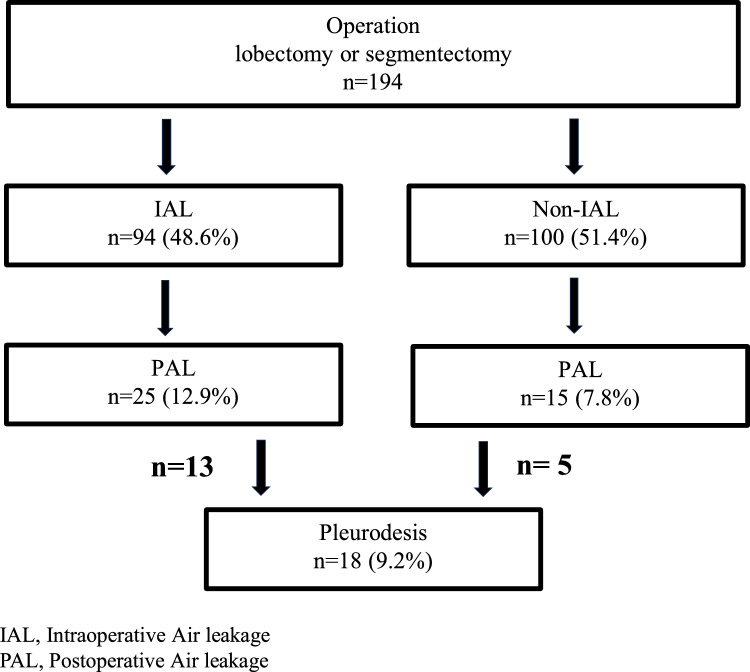
Flowchart of the cases of IAL, PAL, and pleurodesis. IAL, intraoperative air leakage; PAL, postoperative air leakage

Overall, PAL was observed in 40 patients (20.6%), and 18 patients (9.2%) required pleurodesis with OK432 (Fig. 2).

### IAL

A comparison of the IAL and non-IAL groups is shown in Table 2. The IAL group had a significantly longer operative time than the non-IAL group (189.2 vs. 141.6 min; p < 0.001). The IAL group also had a higher Brinkman index of 696.2 (p = 0.0103).

**Table 2:** A comparison of characteristics between the IAL and non-IAL groups

Variables	IAL group (n = 94)	Non-IAL group (n = 100)	P-value
Age (SD)	68.3 (9.72)	70.1 (9.52)	0.1915
Sex			0.3832
Male (%)	63 (67.0)	61 (61.0)	
Female (%)	31 (33.0)	39 (39.0)	
Brinkman index (SD)	696.2 (662.28)	477.1 (503.15)	0.0103
DM (%)	20 (21.3)	14 (14.0)	0.1828
Steroid use (%)	5 (5.3)	7 (7.0)	0.6272
COPD (%)	35 (37.2)	33 (33.0)	0.5368
IP (%)	9 (9.6)	13 (13.0)	0.4521
Approach (%)			0.1877
RATS	18 (19.1)	15 (15.0)	
VATS	14 (14.9)	8 (8.0)	
Open thoracotomy	62 (66.0)	77 (77.0)	
Procedures (%)			0.3828
Lobectomy	63 (67.0)	61 (61.0)	
Segmentectomy	31 (33.0)	39 (39.0)	
Operation time (min) (SD)	189.2 (62.01)	141.6 (38.69)	< 0.0001
Blood loss (ml) (SD)	111 (198.25)	42 (197.29)	0.0021
Staplers used for pulmonary resection (SD)	3.2 (1.68)	2.5 (1.51)	0.0050
Duration of chest tube placement (days) (SD)	2.9 (1.31)	2.2 (1.12)	< 0.0001
Length of hospitalization (days) (SD)	11.3 (12.31)	9.0 (4.23)	0.0816

Data are shown as the mean (standard deviation) or number (percentage)

IAL: intraoperative air leakage; DM: diabetes mellitus; COPD: chronic obstructive pulmonary disease; IP: interstitial pneumonia; RATS: robot-assisted thoracic surgery; VATS: video-assisted thoracic surgery; SD: standard deviation

Other patient characteristics, including COPD, IP, and history of steroid use, did not differ significantly between the two groups. However, the number of staplers used for pulmonary resection was significantly higher in the IAL group than that in the non-IAL group (3.2 vs. 2.5; p = 0.0050). There were no significant differences between the two groups in terms of surgical procedures and approaches. Intraoperative blood loss was significantly higher in the IAL group than in the non-IAL group (111 vs. 42 ml; p = 0.0021). In addition, the duration of chest drainage was longer in the IAL group than in the non-IAL group (2.9 vs. 2.2 days; p < 0.0001). Hospitalization rates did not differ significantly between the two groups.

We analyzed the risk factors for IAL, focusing on the Brinkman index and number of staplers used in pulmonary resection. A cutoff value of 800 for the Brinkman index was determined based on the receiver operating characteristic curve analysis. When comparing a Brinkman index ≥ 800 and a Brinkman index < 800, a univariate analysis demonstrated an odds ratio of 2.2717 (p = 0.009), indicating a statistically significant difference. A multivariate analysis confirmed continued statistical significance, with an odds ratio of 2.072 (p = 0.0229). In terms of the number of staples used, a univariate analysis revealed an odds ratio of 1.2927 (p = 0.005), while a multivariate analysis revealed an odds ratio of 1.2611 (p = 0.01229) (Table 3).

**Table 3:** Risk factors for IAL

Variables	Univariate analysis		Multivariate analysis		
	Odds ratio	P-value	Odds ratio	95% CI	P-value
BI ≥ 800	2.2717	0.009	2.072	1.105–3.938	0.0229
Staplers used for pulmonary resection	1.2927	0.005	1.261	1.261–1.525	0.0123

BI: Brinkman index; CI: confidence interval

### PAL

PAL was observed in 25 (26.6%) and 15 (15%) patients in the IAL and non-IAL groups, respectively (Fig. 2; p = 0.046). A comparison of the characteristics of PAL and non-PAL groups is presented in Table 4. The PAL group had a significantly longer operative time (187.2 min) than the non-PAL group (p = 0.0045). There were no significant differences between the two groups in terms of clinical background information (age, sex, COPD, IP, and history of steroid use). PAL was significantly more common in the lobectomy group than in the segmentectomy group (p = 0.0447), although there was no significant difference between the two groups in terms of the surgical approach or the number of staplers used for pulmonary resection. The PAL group had a significantly longer duration of chest drain placement (p < 0.0001) and hospitalization (p = 0.0097) than did the non-PAL group.

**Table 4:** A comparison of characteristics between the PAL and non-PAL groups

Variables	PAL group (n = 40)	Non-PAL group (n = 154)	P-value
Age (SD)	69.4 (8.62)	69.2 (9.91)	0.9430
Sex			0.1014
Male (%)	30 (75.0)	94 (61.0)	
Female (%)	10 (25.0)	60 (39.0)	
Brinkman index (SD)	632.8 (558.78)	562.8 (596.63)	0.5079
DM (%)	6 (15.0)	28 (18.2)	0.6372
Steroid use (%)	4 (10.0)	8 (5.2)	0.2610
COPD (%)	17 (42.5)	51 (33.1)	0.2678
IP (%)	3 (7.5)	19 (12.3)	0.3899
Approach (%)			0.0720
RATS	11 (27.5)	22 (14.3)	
VATS	6 (15.0)	16 (10.4)	
Open thoracotomy	23 (57.5)	116 (75.3)	
Procedures (%)			0.0447
Lobectomy	31 (77.5)	93 (60.4)	
Segmentectomy	9 (22.5)	61 (39.6)	
Operation time (min) (SD)	187.1 (49.16)	158.8 (56.94)	0.0045
Blood loss (ml) (SD)	92 (197.29)	82 (197.74)	0.7901
Staplers used for pulmonary resection (SD)	2.8 (1.66)	2.9 (1.62)	0.8939
Duration of chest tube placement (days) (SD)	4.2 (1.15)	2.1 (0.91)	< 0.0001
Length of hospitalization (days) (SD)	13.4 (17.27)	9.2 (5.07)	0.0097

Data are shown as the mean (standard deviation) or number (percentage)

PAL: postoperative air leakage; DM: diabetes mellitus; COPD: chronic obstructive pulmonary disease; IP: interstitial pneumonia; RATS: robot-assisted thoracic surgery; VATS: video-assisted thoracic surgery

The risk factors for PAL were also analyzed with respect to surgical procedure and approach. In terms of the surgical procedure (lobectomy or segmentectomy), univariate analysis demonstrated a statistically significant association, with an odds ratio of 2.2592 (p = 0.0447). However, the multivariate analysis showed an odds ratio of 1.9834 (p = 0.0930), which was not statistically significant. In terms of surgical approach (RATS or open thoracotomy), univariate analysis revealed a significant association, with an odds ratio of 2.5217 (p = 0.0382). However, in the multivariate analysis, the odds ratio decreased to 2.1996 and was no longer statistically significant (p = 0.17526) (Table 5).

**Table 5:** Risk factors for PAL

Variables	Univariate analysis		Multivariate analysis		
	Odds ratio	P-value	Odds ratio	95% CI	P-value
Procedure					
Lobectomy	2.2592	0.0447	1.983	0.895–4.752	0.0930
Approach					
RATS/open thoracotomy	2.5217	0.0382	2.200	0.903–4.799	0.0814

CI: confidence interval; PAL: postoperative air leakage; RATS: robot-assisted thoracic surgery

### Pleurodesis

The characteristics of the patients who did or did not require pleurodesis are presented in Table 6. There were no significant differences between the two groups in terms of patient demographics, including the age, sex, Brinkman index, COPD, IP, and history of steroid use. Similarly, the number of staplers used, operative time, and intraoperative blood loss did not differ significantly between groups.

**Table 6:** A comparison of the characteristics between patients who underwent or did not undergo pleurodesis

Variables	Pleurodesis (n = 18)	No pleurodesis (n = 176)	P-value
Age (SD)	71.4 (8.67)	69.0 (9.72)	0.3121
Sex			0.1986
Male (%)	14 (77.8)	110 (62.5)	
Female (%)	4 (22.2)	66 (37.5)	
Brinkman index (SD)	780.3 (618.86)	562.8 (589.81)	0.1282
DM (%)	2 (11.1)	32 (18.2)	0.4523
Steroid use (%)	1 (5.6)	11 (6.3)	0.9073
COPD (%)	9 (50.0)	59 (33.5)	0.1629
IP (%)	1 (5.6)	21 (11.9)	0.4164
Approach (%)			0.0048
RATS	8 (44.0)	25 (14.2)	
VATS	1 (5.6)	21 (11.9)	
Open thoracotomy	9 (50.0)	130 (73.9)	
Procedures (%)			0.0206
Lobectomy	16 (88.9)	108 (61.4)	
Segmentectomy	2 (11.1)	68 (38.6)	
Operation time (min) (SD)	199.8 (48.58)	176.7(48.23)	0.1404
Blood loss (ml) (SD)	91 (198.85)	83 (196.80)	0.8815
Staplers used for pulmonary resection (SD)	3.0 (1.28)	2.7 (1.94)	0.5539
Duration of chest tube placement (days) (SD)	4.7 (1.26)	2.3(1.26)	< 0.0001
Length of hospitalization (days) (SD)	17.3 (9.32)	9.34 (9.11)	0.0003

Data are shown as the mean (standard deviation) or number (percentage)

DM: diabetes mellitus; COPD: chronic obstructive pulmonary disease; IP: interstitial pneumonia; RATS: robot-assisted thoracic surgery; VATS: video-assisted thoracic surgery

However, with respect to the surgical approach, pleurodesis was more commonly performed for RATS cases. Regarding the type of procedure, lobectomy was more frequently performed with pleurodesis (p = 0.0206). Patients who underwent pleurodesis had significantly longer durations of chest drainage (4.7 vs. 2.3 days, p = 0.0001) and hospitalization (17.3 vs. 9.3 days, p = 0.0003) than those who did not undergo pleurodesis.

In terms of the risk of PAL requiring pleurodesis, the surgical procedure (lobectomy or segmentectomy) and approach (RATS or VATS/open thoracotomy) were found to be independent factors. For the surgical procedure (lobectomy or segmentectomy), a univariate analysis revealed an odds ratio of 5.037 (p = 0.0206), which remained statistically significant in the multivariate analysis, with an odds ratio of 4.1611 (p = 0.0350). For the surgical approach (RATS or VATS/open thoracotomy), a univariate analysis revealed an odds ratio of 6.72 (p = 0.0374) for RATS compared with VATS and an odds ratio of 4.622 (p = 0.0055) for RATS compared to open thoracotomy. A multivariate analysis yielded odds ratios of 6.3407 (p = 0.047) for RATS vs. VATS and 3.7134 (p = 0.0185) for RATS vs. open thoracotomy, and both were statistically significant (Table 7).

**Table 7:** Risk factors for prolonged air leakage requiring pleurodesis

Variables	Univariate analysis		Multivariate analysis		
	Odds ratio	P-value	Odds ratio	95% CI	P-value
Procedure					
Lobectomy	5.037	0.0206	4.161	1.094–27.249	0.035
Approach					
RATS/VATS	6.72	0.0374	6.341	1.021–123.328	0.047
RATS/open thoracotomy	4.622	0.0055	3.713	1.255–10.873	0.019

CI: confidence interval; RATS: robot-assisted thoracic surgery; VATS: video-assisted thoracic surgery

## Discussion

In this study, we evaluated the risk factors of IAL and PAL. Although previous studies have investigated PAL, its incidence and associated risk factors have not been examined comprehensively. Our findings demonstrated that IAL occurred in approximately half of the patients during operative procedures for fragile parenchyma. According to Table 2, the IAL group also showed significantly more blood loss and more staples used than the non-IAL group. Indeed, complicated surgery due to adhesion, incomplete lobulation, or other reasons could be the reason for more blood loss, more usage of staples, or more air leakage. IAL can be the result of long-term surgery rather than the reason for it.

One-quarter of the patients with IAL developed PAL, which was 62.5% of the PAL cases, and 45% of the PAL cases required pleurodesis. Pleurodesis was performed in 9.2% of the total number of patients. The number may seem high; however, this is due to our policy of performing early pleurodesis to prevent extended hospital stay and reduce infection.

Although COPD and IP are indicative of lung parenchymal fragility, these patient factors, along with steroid use and diabetes mellitus, were not found to be risk factors for IAL or PAL. However, these findings were not consistent with those of previous studies [12, 13]. The Brinkman index was the only clinical factor that influenced IAL.

Intraoperative factors, such as the type of surgical procedure and approach, were not associated with the risk of IAL. However, significantly more staples were used for pulmonary resection in patients in the IAL group than those in the non-IAL group, suggesting that staple-induced injury to the lung parenchyma may contribute to IAL development. Indeed, the IAL group also showed significantly more blood loss and more staples used than the non-IAL group. These findings suggest that adhesion, incomplete lobulation, or other causes could contribute to greater blood loss, increased staple usage, or more air leakage.

In contrast, PAL occurred in approximately 20% of the patients who underwent anatomical pulmonary resections, and approximately 40% of them did not experience IAL. Unlike the risk factors for IAL, the operative procedure (lobectomy) and RATS approach were risk factors for PAL (Tables 4 and 5).

Although IAL is more reflective of lung fragility and smoking status, as evidenced by the greater number of staples used, PAL seems to be more strongly influenced by the residual thoracic cavity after surgery, as these are more often observed in lobectomy cases. Although segmentectomy typically involves more staples, it is less risky to perform PAL than lobectomy owing to the smaller postoperative residual thoracic cavity space. Although we did not analyze data related to the postoperative residual thoracic cavity content, we hypothesize that lobectomy generally results in a larger residual cavity than segmentectomy because of the greater volume of the resected lung. Specifically, the residual thoracic cavity content following right middle lobectomy (RML) is relatively small. Of the 10 RML cases included in the present study, 2 (20%) experienced PAL; this rate appears to be lower than that of other lobectomy procedures. Furthermore, smoking was not identified as a risk factor for PAL, likely due to the use of pleural reinforcements, such as a polyglycolic acid sheet or fibrin glue, to prevent air leakage when intraoperative findings suggest lung fragility [4, 14–19].

Considering its significance in patients, pleurodesis was the focus of the present study. Based on our results related to the risk factors and patient diagrams, pleurodesis would be more related to PAL than IAL because of the patients who underwent pleurodesis; only 5 patients (28%) experienced IAL. This suggests that the effort by surgeons to repair the IAL during surgery contributed to the reduction in PAL and pleurodesis. The requirement for pleurodesis was significantly higher in RATS cases than in either VATS or open thoracotomy cases, possibly because this study included many patients in the early stages of RATS implementation. As the surgeons were not yet familiar with suturing with RATS, this may have resulted in inadequate IAL repair and more cases of pleurodesis. The repair methods did not differ between the approaches. We also implemented an early pleurodesis policy in these cases. In patients with an elevated risk of IAL, the use of buttressed staple lines has been reported to be effective in preventing air leakage from stapler-related sites [4, 16, 17]. Although the details of the location of the IAL are not known, air leakage typically occurs at a staple-related site, such as a staple hole. Therefore, pulmonary resection using a buttress automatic suturing device may be effective when staple-related air leakage is anticipated.

Several limitations associated with the present study warrant mention. First, it was a single-institution retrospective study with a relatively small sample size. Second, detailed records specifying whether the surgery was terminated with complete air leakage closure or with minor residual air leakage that was anticipated to resolve over time were unavailable. PAL was evaluated primarily using postoperative medical records, resulting in cases with no IAL but detectable PAL or with repaired IAL that persisted postoperatively. Third, although most surgeons were experienced thoracic surgeons, some procedures were performed by surgeons in training, which introduced the possibility of surgeon-related bias due to air leakage resulting from technical inexperience.

## Conclusion

The clinical risk factors differed between IAL and PAL. Since the majority of PAL cases involved IAL and approximately half of the PAL cases required pleurodesis, attention should be paid to the prevention of IAL, especially during stapling procedures in the lung parenchyma. Further prospective studies are required to clarify the incidence of IAL and the relationship between IAL and PAL.

## Meeting Presentation

This manuscript was presented at the International Association for the Study of Lung Cancer 2024 World Conference on Lung Cancer (September 9–12, 2023).
